# Comparative patterns in taxonomic and functional spider diversities between tropical vs. temperate forests

**DOI:** 10.1002/ece3.6907

**Published:** 2020-10-30

**Authors:** Kaïna Privet, Julien Pétillon

**Affiliations:** ^1^ G‐Tube (Géoarchitecture: territoires, urbanisation, biodiversité, environnement) ‐ EA 7462 Univ Rennes Rennes France; ^2^ CNRS Ecobio (Écosystèmes, biodiversité, évolution) – UMR 6553 Univ Rennes Rennes France

**Keywords:** alpha diversity, Araneae, deciduous trees, France, French Guiana, functional diversity, intensive sampling

## Abstract

High diversity in tropical compared to temperate regions has long intrigued ecologists, especially for highly speciose taxa like terrestrial arthropods in tropical rainforests. Previous studies showed that arthropod herbivores account for much tropical diversity, yet differences in the diversity of predatory arthropods between tropical and temperate systems have not been properly quantified. Here, we present the first standardized tropical–temperate forest quantification of spider diversities, a dominant and mega‐diverse taxon of generalist predators. Spider assemblages were collected using a spatially replicated protocol including two standardized sampling methods (vegetation sweep netting and beating). Fieldwork took place between 2010 and 2015 in metropolitan (Brittany) and overseas (French Guiana) French territories. We found no significant difference in functional diversity based on hunting guilds between temperate and tropical forests, while species richness was 13–82 times higher in tropical versus temperate forests. Evenness was also higher, with tropical assemblages up to 55 times more even than assemblages in temperate forests. These differences in diversity far surpass previous estimates and exceed tropical–temperate ratios for herbivorous taxa.

## Graphical abstract

To deepen our knowledge of latitudinal gradient in predatory arthropods diversity, we conducted an unpreceded quantification of tropical and temperate spider diversity difference using a standardized statistical rarefaction approach. We did not find any differences in functional diversity among biomes, while the ratio of spider diversity we measured was up to 30 times what was previously proposed for predators through indirect comparisons (result consistent between diversity metrics and sampling methods). This original result calls for a reanalysis of the relationship between predatory arthropod diversity, plant species richness, and phylogenetic diversity.

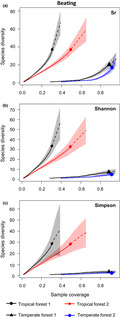

## 1 INTRODUCTION

The latitudinal gradient of diversity, that is increase in species richness with decreasing latitude, has long been recognized by the scientific community (Pianka, 1966). Gradients of diversity in various arthropod taxa from tropical to temperate and even polar ecosystems are well documented through meta‐analyses (Willig et al., 2003; but see Hillebrand, 2004). Arthropods were particularly studied in tropical rainforests (the species richest terrestrial ecosystem: Miller et al., 2002) where nearly 1.5 million tropical arthropod species are currently described out of an estimated number of 2 to 7 million tropical arthropod species (Hamilton et al., 2010; Stork, 2017). Herbivorous arthropod assemblages have been extensively studied in both tropical and temperate forests with studies of diversity, species richness per plant, host specificity, and herbivory pressure. Herbivore arthropod diversity, as well as rate of herbivory, are considered higher in tropical systems compared to temperate counterparts (Lim et al., 2015; Peguero et al., 2017), though evidence of greater host specificity is still controversial (Novotny, 2006; Peguero et al., 2017). Such gradients in herbivore diversity can be explained by underlying plant diversity, herbivore diet specialization, and plant defense. Less well studied is the possible role of natural enemies (i.e., predators and parasitoids) on herbivore arthropod diversity (Björkman et al., 2011). Latitudinal gradients in the diversity of omnivore arthropods have also been studied, mainly in ants for which assemblages are clearly species richer in tropical versus temperate systems (Jaffre et al., 2007; Jeanne, 1979). For example, canopy assemblages of ants from tropical forests are estimated to be 4 times richer than those from temperate forests (Jaffre et al., 2007). Although ants are considered the main predatory arthropods in tropical rainforests (Floren et al., 2002), they complete a large variety of functional roles (Dejean & Corbara, 2003), and their diversity thus does not reflect the diversity of predatory arthropod taxa.

Few studies have examined the latitudinal gradient of predatory arthropod diversity, while other macro‐ecological patterns were investigated in these taxa (e.g., for spiders: Arvidsson et al., 2016; Finch et al., 2008; Kozlov et al., 2015; Pitta et al., 2019; Ysnel et al., 2008). To date, most of the studies focused on predation pressure, for example, highlighting that predation pressure increases when latitude decreases (Andrew & Hughes, 2005; Novotny, 2006; Rodríguez‐Castañeda, 2013), but sometimes remains constant (Cardoso et al., 2011; Zhang & Adams, 2011). Lacking are studies that directly compare the diversity of tropical versus temperate for predatory arthropods (Schuldt et al., 2013), despite their strong contribution to ecosystem diversity and functioning (Björkman et al., 2011).

To the best of our knowledge, only one study has evaluated and quantified the difference of tropical versus temperate diversity in predatory arthropods. It was conducted by Basset et al. (2012) who performed a comparison of tropical and temperate forests for different trophic guilds based on data obtained independently, using different sampling protocols. They estimated that differences in predatory arthropod diversity between tropical and temperate ecosystems should be in the same range as those for herbivorous arthropods, with tropical assemblages being 2 to 8.4 times more diverse compared to temperate forest (Basset et al., 2012). Although spiders constitute a relevant model taxon to compare predatory arthropods between temperate and tropical regions, this ratio has never been tested nor confirmed using spiders only. They are indeed one of the few taxa, if any other, that is exclusively, except for one species and occasional plant consumption by few other species (see the recent review by Nyffeler et al., 2016), composed by predatory species (Birkhofer & Wolters, 2012).

We present here the first standardized tropical–temperate quantification for vegetation‐dwelling spider diversity using the same spatially and method‐replicated sampling protocol. More specifically, we compared patterns of both taxonomic and functional diversities as they bring complementary information on ecological and evolutionary processes (Tucker et al., 2018). We first expected (a) correlated patterns between taxonomic and functional diversities (as previously documented in plants and vertebrates: see Tucker & Cadotte, 2013, but also in arthropods, e.g., Birkhofer et al., 2015 and Ridel et al., 2020), (b) consistently (much) more diversity and evenness in tropical compared to temperate forests due a longer time of diversification processes leading to more species and traits co‐existence, and (c) an order of magnitude between temperate and tropical forests in the same range than what previously reported for other arthropods, that is, diversity and evenness around 8 times higher in tropical compared to temperate forests.

## 2 METHODS

### 2.1 Study sites

Tropical and temperate sampling were replicated in both locations and sampling methods, to increase generalization power (Willis & Whittaker, 2002).

The two replicated tropical sites were two nature reserves in French Guiana (South America) sharing similar climates: La Trinité Reserve (76,900 ha; 4°35′2″N, 53°18′1″W) and Nouragues Reserve (105,000 ha; 4°04′18″N, 52°43’57″W). These sites are seasonally flooded rainforests with representative vegetation of the primary lowland rainforest, with few inclusions of palmetto–swamp forests, liana forests, and bamboo forests. Both forests were sampled during the rainy season, considered as the period of maximum diversity in tropical forests (e.g., Gasnier & Höfer, 2001). La Trinité and Les Nouragues were hereafter called tropical forest one and tropical forest two, respectively.

Temperate sites were in two forests preserves of mixed hardwood forests in Brittany (Western France): the forest of the military camp of Saint‐Cyr‐Coëtquidan (2,000 ha; 47°57′50″N, 2°11′30″W) and the state‐owned forest of Rennes (3,000 ha; 48°11′53″N, 1°33′22″W). The vegetation of these forests is representative of many temperate forests with some shrubby species, small trees, and climbing plants. Only forest types dominated by native deciduous trees were sampled. Both forests were sampled in summer, the period estimated to have maximal spider diversity (see Hsieh & Linsenmair, 2012). Saint‐Cyr‐Coëtquidan and Rennes were hereafter called temperate forest one and temperate forest two, respectively.

While the actual sampled area was similar in all four forests, we consider the size of studied forests to be a confounding factor and an intrinsic part of the difference between tropical and temperate forests, as there are anyway no temperate forests as big as the Amazonian forest, to which the two tropical Nature Reserves sampled here belong. Tree species richness is also an intrinsic difference between the forests in each biome, with around 150 species in both Trinité and Les Nouragues tropical forests (see Guitet et al., 2018 and Poncy et al., 1998, respectively), and 10 times less in Rennes and Coëtquidan temperate forests (V. Jung comm. pers. and Morel et al., 2020, respectively).

### 2.2 Sampling and Identification

We developed a quasi‐optimal protocol (sensu Malumbres‐Olarte et al., 2017 who defined it as a “standardized protocol that may not be optimal for any specific site alone.”) designed for short and intensive surveys. In each forest, we used two surface‐standardized active sampling methods highly efficient for vegetation‐dwelling spiders (Coddington et al., 2009): beating and sweep netting. Vegetation beating was conducted in 9 × 9 m quadrats where the vegetation was beaten with a stick over a beating tray to a height of 2.5 m. In each forest, 12 randomly selected quadrats were conducted by four people in two duos concurrently (six quadrats per duo). Sweep netting was carried out with a sweep net along 20 m long and one‐meter‐wide (arm length plus sweep handle) transects. Twelve randomly selected transects were conducted in each forest by the same two persons.

All quadrats and transects were carried out in visually homogeneous areas of each forest that differed between methods. Tropical forest one was sampled 3–7 December 2010, tropical forest two, 6–15 December 2013, temperate forest one, 15–16 June 2015, and temperate forest two, 22–23 June 2015.

Temperate adult spiders were sorted and identified to species, while tropical adult spiders were identified to morphospecies because of a lack of taxonomic knowledge in the tropics (Scharff et al., 2003). Whenever possible, males and females were matched together and grouped into one single morphospecies. All specimens were identified by the authors and stored at the University of Rennes 1, France.

### 2.3 Data analysis

Because limited information for tropical spiders, functional metrics was based on abundance of family hunting guilds only (Cardoso et al., 2011), using FD R package on the Gower dissimilarity matrix with a Cailliez correction (Laliberté et al., 2014).

The difference between functional diversity and evenness observed in each biome with each sampling technique was assessed using a mixed linear model with a Gaussian distribution. Functional diversity and evenness were the response variables, and biome and site were the predictors (respectively, fixed and random factors). Normality of results was checked using diagnostic plots.

We standardized the comparison of taxonomic diversity between the four forests by using species rarefaction and extrapolation curves based on sample coverage (Chao et al., 2014). Analyses were completed using the R‐based iNEXT package (Chao et al., 2014) with R Software (R Development Core Team, 2018) on summed species abundances over the 12 replicates per method and per site. iNEXT function was configured at 40 knots and 200 bootstraps replications. 95% confidence intervals (CI) were calculated for the three measures of species diversity (species richness, Shannon, and Simpson diversity indices) within overlap of CI used to indicate a significant difference at a level of 5% among the expected diversities (Chao et al., 2014). Diversities were compared at the same sample coverage (named “base coverage”), following Chao et al. (2014), allowing for a standardized comparison of spider assemblage diversity between biomes despite differences in forest areas. Comparisons were conducted at 38.8% sample coverage for beating and at 60% sample coverage for sweep netting.

## 3 RESULTS

A total of 2,846 individuals belonging to 202 (morpho‐)species were collected (see detailed taxonomic list: Table S1).

No significant differences were found between models of functional diversity based on beating sampling (*t* = −0.082, *df* = 1.3, *p* = .529) or based on sweep netting *t* = 26.06, *df* = 1.9, *p* = .195), indicating an absence of a biome effect. The same was found for functional evenness, that is, no significant effect of biome on this metric by beating (*t* = 0.79, *df* = 1.92, *p* = .515) and by sweep netting (*t* = 2.84, *df* = 1.96, *p* = .107).

**Figure 1:**
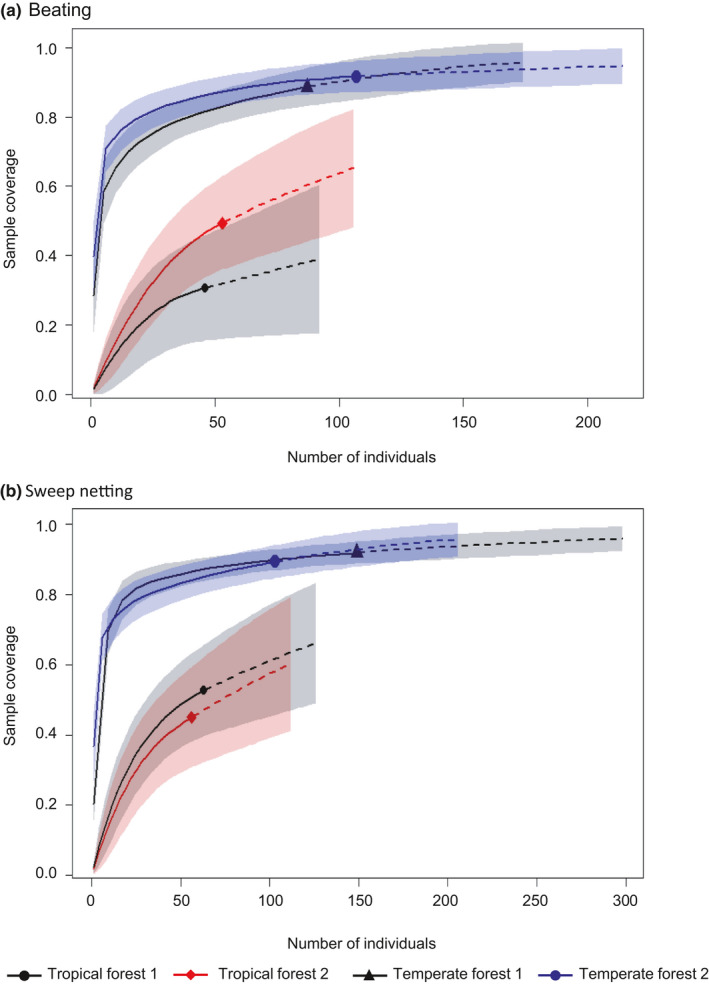
Sample coverage for rarefied samples (solid line) and extrapolated samples (dashed line) as a function of sample size for spider samples collected by (a) beating and (b) sweep netting in tropical rainforests one and two (La Trinité and Les Nouragues) and the temperate deciduous forests one and two (Coëtquidan and Rennes). The 95% confidence intervals are represented in light color and were obtained by a bootstrap method (Chao et al., 2014) based on 200 replications. Reference samples in each forest are denoted by solid markers. For comparison, all curves were extrapolated up to double its reference sample size. The numbers in parentheses are the sample coverage and the number of individuals for reference samples

Based on rarefaction, the sample coverage was nearly two times higher in temperate forests for the two sampling methods and almost any sample size (i.e., number of individuals; Figure 1a,b). When comparing samples at the same effective sample size for both methods, sample coverage was about 90% in temperate and between 30% and 53% in tropical forests. Thus, even though the same standardized protocol was used in both biomes, temperate samples were two to three times more complete than tropical ones. Based on the extrapolation for both sampling methods, when the sample size was doubled, the sample coverage increased by three to seven percent for temperate forests and by nine to 16% in tropical ones (Figure 1a, b).

**Figure 2:**
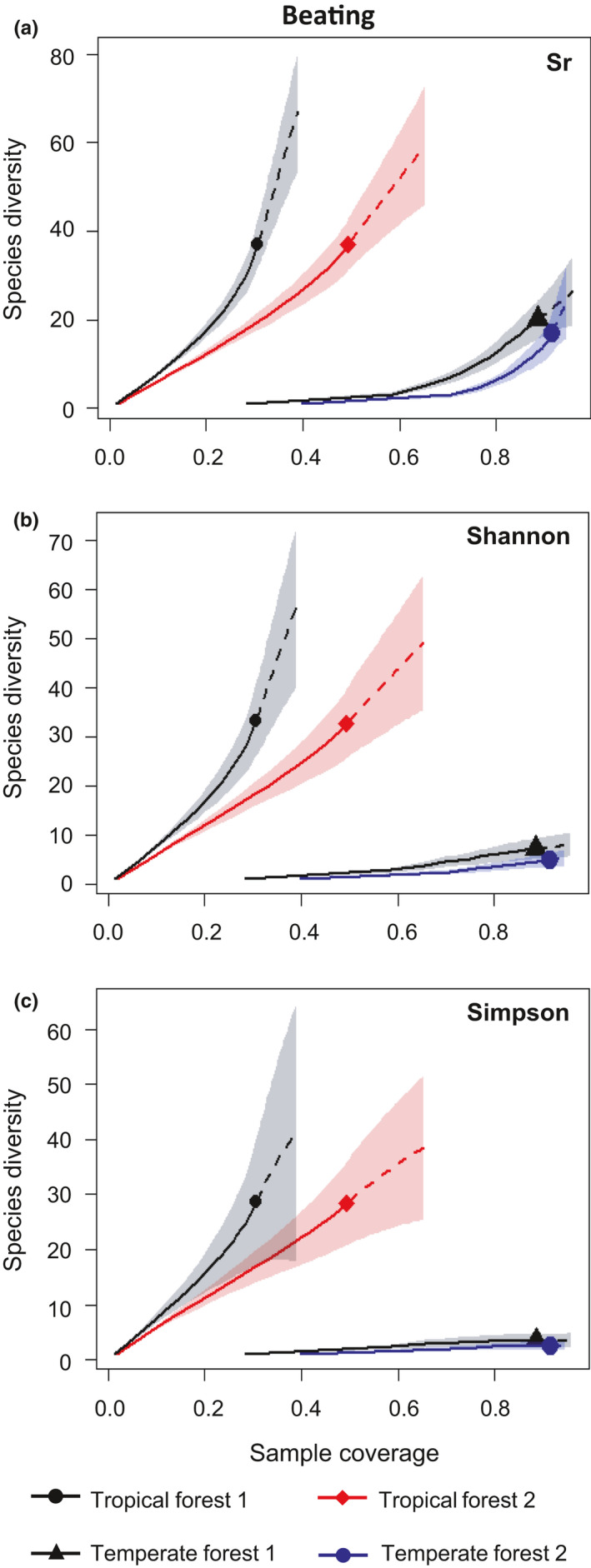
Comparison of the coverage‐based rarefaction (solid line) and extrapolation (dashed line) of spider (a) species richness (Sr), (b) Shannon diversity and (c) Simpson diversity collected by beating in tropical rainforests one and two (La Trinité and Les Nouragues) and the temperate deciduous forests one and two (Coëtquidan and Rennes). The 95% confidence intervals are represented in light color and were obtained by a bootstrap method (Chao et al., 2014) based on 200 randomizations. Reference samples in each forest are denoted by solid markers. For comparison, all curves were extrapolated up to double its reference sample size. The numbers in parentheses are the sample coverage and the observed diversity indices (species richness, Shannon, or Simpson) for each reference sample

**Figure 3:**
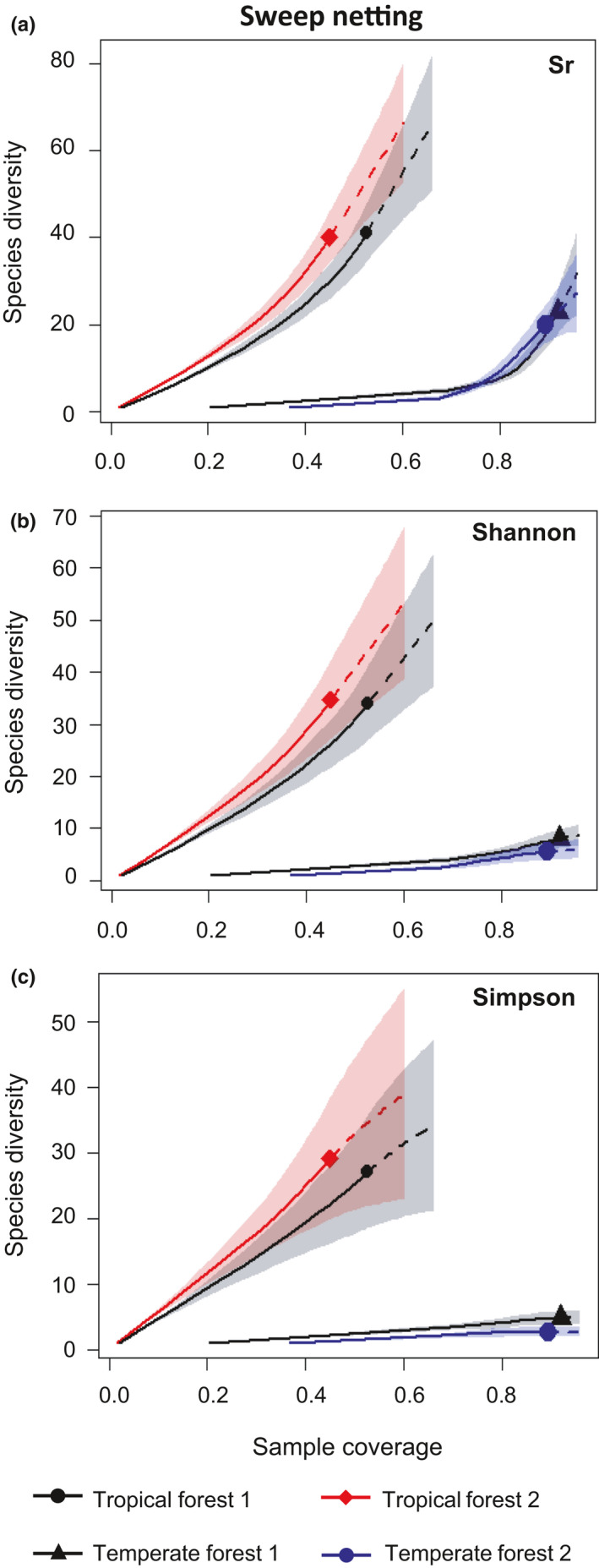
Comparison of the coverage‐based rarefaction (solid line) and extrapolation (dashed line) of spider (a) species richness (Sr), (b) Shannon diversity, and (c) Simpson diversity collected by sweep netting in tropical rainforests one and two (La Trinité and Les Nouragues) and the temperate deciduous forests one and two (Coëtquidan and Rennes). The 95% confidence intervals are represented in light color and were obtained by a bootstrap method (Chao et al., 2014) based on 200 replications. Reference samples in each forest are denoted by solid markers. All curves were extrapolated up to double its reference sample size. The numbers in parentheses are the sample coverage and the observed diversity indices (species richness, Shannon, or Simpson) for each reference sample

When comparing coverage‐based diversities of tropical and temperate forests at the same sample coverages, confidence bands of the replicated sites of tropical and temperate forests did not overlap for either beating or sweep netting (Figures 2 and 3). Thus, tropical spider assemblages were highly and significantly more diverse than temperate ones for any sample coverage, sampling method, and diversity indices used (see detailed results below).

Beating and sweep netting consistently showed the same patterns. Tropical spider assemblages were 12.9 to 81.6 times species richer than temperate ones (Figures 2 and 3). Difference in diversity between biomes was also significant for Shannon diversity and for Simpson diversity. Shannon diversity was 11.6 to 54.6 times higher in tropical assemblages than in temperate counterparts, and Simpson diversity was 10.4 to 40.4 times higher in tropical assemblages (Figures 2 and 3).

## 4 DISCUSSION

Using a spatially and method‐replicated protocol, we found that the taxonomic diversity of spiders was much higher in tropical forests compared to temperate forests (with consistent patterns for all diversity metrics and for the two sampling methods), when no difference was detected for functional diversity.

The fact that functional diversity was not differing among biomes can indicate either similar levels of or balanced effects of both habitat filtering and interspecific competition (Fichaux et al., 2019), which would be especially interesting at such a large spatial scale. Phylogenetic diversity can also be an interesting side of diversity, which might not be correlated with taxonomic and functional diversities as well (Tucker et al., 2018). Cardoso et al. (2011) also suspected spider taxonomic diversity to be higher in the tropics, but with species functionally redundant, which was supported by Schuldt et al. (2013) who compared tropical (China) and temperate (Germany) spider assemblages. The functional diversity was yet based on hunting guilds only in this study and should be computed with other traits in the future when they will be available at large spatial scales (Lowe et al., 2020).

Our study showed that, with the same level of sample coverage, species richness of tropical forest spiders was 13–82 times higher than temperate species richness. This magnitude of difference is much greater than expected (i.e., two to eight times more than in Basset et al., 2012). The comparison of evenness also revealed that the spider assemblages we sampled in tropical forests were also up to 55 times more even than in temperate forests. Weighted measures of diversity (i.e., species evenness and species dominance) are known to provide more comprehensive views of patterns of taxonomic diversity (Willig et al., 2003). Diversity metrics responded in the same way than species richness (and consistently between sampling methods), which confirms that spider diversity was up to 30 times higher than what was previously proposed for predatory arthropods through indirect comparisons.

Although several methodological factors could influence the difference in ratios between Basset et al. (2012) and this study, and among them the indirect comparison used by Basset et al. (2012), the strata sampled (understory here vs. soil to canopy for Basset et al., 2012), and the species richness estimation methods (a large range of different estimators in Basset et al., 2012) but without considering sample coverage), we still argue that predatory arthropods are proportionally more diverse in tropical compared to temperate forests than other taxa, like, for example, herbivore arthropods and plants. The global difference in diversity between tropical and temperate ecosystems is indeed partly explained by both plant species richness and plant phylogenetic diversity (Dinnage et al., 2012), which suggests that the diversity of predatory arthropods also mirrors plant diversity. Interestingly, the values of tree diversity of our study sites are comparable to those of other temperate and neotropical forests (see e.g., Brokaw & Busing, 2000) and also fit to previous estimations of 5–10 times more plant species per hectare in tropical compared to temperate areas (Barthlott et al., 1996). Hence, the 13–82 times higher species richness of spiders in tropical rainforest would be vastly higher than the actual difference in tree diversity between the same pairs of forests. Thus, spiders would be 1.2 to 16 times proportionally richer than plants in tropical compared to temperate systems. These results suggest that the relationship between spider and plant diversity in tropical forest would not be one‐to‐one as it was previously estimated for all trophic level arthropods (Basset et al., 2012; Dinnage et al., 2012). The ratio between plant and spider diversity in tropical forests compared to temperate forests could be higher due to a wider diet of spiders in tropical versus temperate forests (see Birkhofer & Wolters, 2012 for further information). Lastly, vegetation structure, known to affect spider diversity (see e.g., Hurd & Fagan, 1992), could also have played a role in shaping differences of species richness between biomes. But understory structure, that was not quantified here, did not look so different between (primary) tropical and (secondary) temperate forests, even possibly higher in the latter (K. Privet & J. Pétillon, pers. observations).

Finally, intensive sampling in tropical regions is often limited in time and replication, which potentially induces biases such as random effects and particular local conditions. We are aware that our design would have benefited from additional replication, but there is also a risk to increase intratreatment variance by doing so in a single design (Oksanen, 2001). Therefore, we encourage tropical researchers to continue sampling spider diversity, using this or other standardized sampling protocols, in paired comparisons of tropical versus temperate forests to infer on eco‐evolutionary drivers of biodiversity patterns at large spatial scales.

## CONFLICT OF INTEREST

None declared.

## AUTHOR CONTRIBUTION

**Kaïna Privet:** Conceptualization (supporting); Formal analysis (lead); Writing‐original draft (lead); Writing‐review & editing (supporting). **Julien Pétillon:** Conceptualization (lead); Data curation (lead); Formal analysis (supporting); Funding acquisition (lead); Project administration (lead); Writing‐original draft (supporting); Writing‐review & editing (lead).

## Supporting information

Table S1

## Data Availability

Basic data can be found as part of an Electronic Supplementary Material. More details are available with the last author upon reasonable request.
