# Supplementary material for: Comparative patterns in taxonomic and functional spider diversities between tropical vs. temperate forests
Source: Ecol Evol. 2020 Oct 30;10(23):13165–72. doi: 10.1002/ece3.6907 (PMC7713944; doi:10.1002/ece3.6907)
Supplement: Supplementary file 1 — Table S1 [file ECE3-10-13165-s001.docx]

**Supporting information**

**TABLE S1:** List and abundances of species collected during this study. Part of the spiders collected in tropical forests were unknown and only tropical spiders belonging to the family Salticidae were identified to the species level.

| **Forest** | **Species** | **Abundance** |
| --- | --- | --- |
| **Temperate forest 1  (Saint-Cyr-Coëtquidan)** | Anelosimus vittatus | 1 |
|  | Araneus diadematus | 1 |
|  | Ballus chalybieus | 2 |
|  | Clubiona comta | 4 |
|  | Clubiona terrestris | 1 |
|  | Diaea dorsata | 2 |
|  | Dipoena melanogaster | 1 |
|  | Enoplognatha ovata | 102 |
|  | Episinus maculipes | 1 |
|  | Gongylidium rufipes | 3 |
|  | Hypomma cornutum | 2 |
|  | Linyphia hortensis | 2 |
|  | Mangora acalypha | 1 |
|  | Metellina mengei | 31 |
|  | Metellina meriane | 4 |
|  | Neottiura bimaculata | 2 |
|  | Neriene peltata | 1 |
|  | Neriene radiata | 1 |
|  | Oedothorax fuscus | 2 |
|  | Paidiscura pallens | 2 |
|  | Parasteatoda simulans | 2 |
|  | Pelecopsis parallela | 1 |
|  | Philodromus albidus | 2 |
|  | Philodromus rufus | 1 |
|  | Platnickina tincta | 2 |
|  | Porrhomma microphtalmum | 1 |
|  | Tenuiphantes flavipes | 33 |
|  | Tenuiphantes zimmermanni | 14 |
|  | Tetragnatha montana | 4 |
|  | Theridion varians | 2 |
|  | Theridiosoma gemmosum | 4 |
|  | Zilla dioidia | 4 |
| **Temperate forest 2  (Rennes)** | Anelosimus vittatus | 1 |
|  | Araneus diadematus | 1 |
|  | Batyphantes gracilis | 5 |
|  | Ceratinella scabrosa | 1 |
|  | Clubiona brevipes | 1 |
|  | Diaea dorsata | 1 |
|  | Dipoena melanogaster | 1 |
|  | Enoplognatha ovata | 127 |
|  | Erigone atra | 1 |
|  | Erigone dentipalpis | 2 |
|  | Hypomma cornutum | 1 |
|  | Linyphia hortensis | 1 |
|  | Metellina mengei | 17 |
|  | Neriene peltata | 2 |
|  | Nigma puella | 1 |
|  | Oedothorax fuscus | 1 |
|  | Oedothorax retusus | 1 |
|  | Paidiscura pallens | 7 |
|  | Parasteatoda lunata | 2 |
|  | Parasteatoda simulans | 4 |
|  | Philodromus albidus | 1 |
|  | Philodromus dispar | 3 |
|  | Philodromus praedatus | 1 |
|  | Platnickina tincta | 14 |
|  | Tenuiphantes flavipes | 5 |
|  | Tenuiphantes tenuis | 1 |
|  | Tenuiphantes zimmermanni | 2 |
|  | Tetragnatha montana | 1 |
|  | Tetragnatha obtusa | 2 |
|  | Theridion varians | 1 |
|  | Walckenaria vigilax | 1 |
| **Tropical forest 1  (La Trinité)** | AnaA | 1 |
|  | AnaB | 1 |
|  | AnyA | 1 |
|  | AnyH | 1 |
|  | AraAP | 1 |
|  | AraAR | 1 |
|  | AraAT | 1 |
|  | AraC | 1 |
|  | AraD | 1 |
|  | AraE | 1 |
|  | AraG | 1 |
|  | AraH | 1 |
|  | AraI | 1 |
|  | AraJ | 3 |
|  | AraL | 1 |
|  | AraP | 1 |
|  | AraV | 1 |
|  | CluA | 1 |
|  | Cobanus sp3 | 1 |
|  | CorA | 1 |
|  | CorH | 1 |
|  | CorJ | 1 |
|  | CorK | 1 |
|  | CorL | 1 |
|  | CorM | 1 |
|  | Corythalia sp4 | 1 |
|  | Cylistella sp1 | 1 |
|  | Erica eugenia | 1 |
|  | Gen9 sp1 | 1 |
|  | Hypaeus porcatus | 1 |
|  | Hypaeus sp2' | 3 |
|  | Hypaeus taczanowskii | 10 |
|  | LinA | 1 |
|  | LinE | 1 |
|  | LinI | 1 |
|  | Lyssomanes longipes | 2 |
|  | Lyssomanes nigropictus | 1 |
|  | Lyssomanes sp3 | 1 |
|  | Mago longidens | 1 |
|  | MimA | 4 |
|  | Noegus niveomarginatus | 4 |
|  | Noegus sp1 | 2 |
|  | Noegus sp11 | 1 |
|  | Noegus sp5 | 1 |
|  | OxyoA | 1 |
|  | OxyoB | 2 |
|  | PholA | 3 |
|  | PisC | 1 |
|  | PisE | 1 |
|  | PisF | 1 |
|  | PisG | 1 |
|  | PisI | 1 |
|  | Scopocira cf tenella | 3 |
|  | ScyA | 4 |
|  | ScyB | 1 |
|  | ScyC | 1 |
|  | Soesilarishius sp1 | 1 |
|  | TetF | 1 |
|  | TherAA | 2 |
|  | TherAF | 1 |
|  | TherAG | 1 |
|  | TherAH | 1 |
|  | TherAI | 1 |
|  | TherAJ | 1 |
|  | TherAK | 1 |
|  | TherAL | 1 |
|  | TherAQ | 1 |
|  | TherB | 10 |
|  | TherC | 2 |
|  | TherD | 6 |
|  | TherE | 1 |
|  | TherG | 3 |
|  | TherH | 2 |
|  | TherI | 1 |
|  | TherJ | 2 |
|  | TherK | 2 |
|  | ThoAD | 1 |
|  | ThoAE | 1 |
|  | ThoAF | 2 |
|  | ThoAK | 1 |
|  | ThoB | 1 |
|  | ThoC | 10 |
|  | ThoD | 1 |
|  | ThoE | 6 |
|  | ThoF | 1 |
|  | ThoH | 6 |
|  | ThoI | 5 |
|  | ThoJ | 1 |
|  | ThoN | 1 |
|  | ThoO | 1 |
|  | ThoP | 5 |
|  | ThoQ | 1 |
|  | UloA | 2 |
|  | UloD | 4 |
|  | UloJ | 1 |
|  | UloK | 1 |
| **Tropical forest 2  (Les Nouragues)** | Ana2 | 1 |
|  | Any1 | 1 |
|  | Any12 | 1 |
|  | Any13 | 1 |
|  | Ara23 | 2 |
|  | Ara25 | 6 |
|  | Ara33 | 4 |
|  | Ara40 | 2 |
|  | Ara53 | 1 |
|  | Ara58 | 2 |
|  | Ara59 | 1 |
|  | Ara60 | 1 |
|  | Ara61 | 1 |
|  | Ara62 | 1 |
|  | Ara63 | 1 |
|  | Ara66 | 1 |
|  | Ara72 | 2 |
|  | Chira sp1 | 1 |
|  | Cor3 | 5 |
|  | Cor5 | 1 |
|  | Cor7 | 1 |
|  | Cotinusa sp2 | 1 |
|  | Cylistella sp1 | 7 |
|  | Gen20 sp1 | 1 |
|  | Hypaeus sp2' | 1 |
|  | Hypaeus taczanowskii | 5 |
|  | Lin5 | 1 |
|  | Mago longidens | 4 |
|  | Neogus sp10 | 1 |
|  | Nest1 | 1 |
|  | Oon5 | 2 |
|  | Sidusa sp1 | 1 |
|  | Soesilarishius aurifrons | 1 |
|  | Soesilarishius ruizi | 1 |
|  | Soesilarishius sp1 | 1 |
|  | Syn3 | 1 |
|  | Syn7 | 1 |
|  | Tet10 | 1 |
|  | Tet4 | 2 |
|  | Tet7 | 1 |
|  | Tet9 | 1 |
|  | Ther10 | 1 |
|  | Ther16 | 1 |
|  | Ther18 | 3 |
|  | Ther21 | 1 |
|  | Ther55 | 2 |
|  | Ther56 | 1 |
|  | Ther57 | 1 |
|  | Ther59 | 1 |
|  | Ther60 | 2 |
|  | Ther61 | 1 |
|  | Ther62 | 1 |
|  | Ther63 | 1 |
|  | Ther64 | 1 |
|  | Ther65 | 1 |
|  | Therio2 | 1 |
|  | Tho15 | 1 |
|  | Tho16 | 1 |
|  | Tho17 | 1 |
|  | Tho18 | 2 |
|  | Tho19 | 1 |
|  | Tho2 | 1 |
|  | Tho22 | 1 |
|  | Tho3 | 5 |
|  | Tho9 | 4 |
|  | ThoA | 1 |

(b)
